# Effects of circadian rhythm on Narcotrend index and target-controlled infusion concentration of propofol anesthesia

**DOI:** 10.1186/s12871-021-01445-z

**Published:** 2021-09-06

**Authors:** Jiang-hua Shen, Min Ye, Qian Chen, Yan Chen, Hai-lin Zhao, Ameena Khan, Bin Yi, Jiao-lin Ning, Kai-zhi Lu, Jian-teng Gu

**Affiliations:** 1 Department of Anesthesiology, Southwest Hospital, Third Military Medical University (Army Medical University), No.30 Gaotanyan Road, Shapingba district, Chongqing, China; 2 Division of Anesthetics, Pain Medicine and Intensive Care, Department of Surgery and Cancer, Faculty of Medicine, Imperial College London, Chelsea & Westminster Hospital, London, UK

**Keywords:** Circadian rhythm, Propofol, Target controlled infusion, Depth of anesthesia

## Abstract

**Background:**

The effects of circadian rhythms on drug metabolism and efficacy are being increasingly recognized. However, the extent to which they affect general anesthesia remains unclear. This study aims to investigate the effects of circadian rhythms on anesthetic depth and the concentrations of propofol target-controlled infusion (TCI).

**Methods:**

Sixty patients undergoing laparoscopic surgeries were sequentially assigned to four groups. Group ND (n = 15): Propofol TCI with Narcotrend monitor during the day (8:00–18:00), Group NN (n = 15): Propofol TCI with Narcotrend monitor during the night (22:00–5:00), Group CLTD (n = 15): Propofol closed-loop TCI guided by bispectral index (BIS) during the day (8:00–18:00), Group CLTN (n = 15): Propofol closed-loop TCI guided by BIS during the night (22:00–5:00). The Narcotrend index, mean arterial pressure (MAP) and heart rate (HR) were compared between group ND and NN at 7 time points, from 5 min before induction to the end of operation. The propofol TCI concentrations, MAP and HR were compared between group CLTD and CLTN at 7 time points, from 5 min after induction to the end of operation.

**Results:**

The Narcotrend index, MAP, and HR in group NN were lower than those in group ND from the beginning of mechanical ventilation to the end of operation (*p* < 0.05). The propofol TCI concentrations in group CLTN were lower than those in group CLTD from the beginning of operation to the end of operation (*p* < 0.05).

**Conclusion:**

Circadian rhythms have a significant effect on the depth of anesthesia and drug infusion concentrations during propofol TCI. When using general anesthesia during night surgery, the propofol infusion concentration should be appropriately reduced compared to surgery during the day.

**Trial registration:**

The present study was registered on the ClinicalTrials.gov website (NCT02440269) and approved by the Medical Ethics Committee of Southwest Hospital of Third Military Medical University (ethics lot number: 2016 Research No. 93). All patients provided informed written consent to participate in the study.

## Background

Circadian rhythms, changes of behavioral or physiological activities with a cycle of about 24 h due to the earth’s rotation, are controlled by the suprachiasmatic nucleus in the hypothalamus. It is generally believed that circadian rhythms evolved independently in a convergent process to adapt to and use the daily geophysical cycle for maximum survival and competitive advantage [1]. Circadian rhythms have been proven to influence a series of physiological behaviors including sleep arousal, metabolism, and reproduction, by regulating mammalian hormone generation and degradation [2]. Furthermore, the pharmacokinetics and pharmacodynamics of various drugs used in anesthesia are impacted by the circadian rhythms [3, 4].

Target-controlled infusion (TCI) is a widely used standardized infusion system that can provide appropriate concentrations of anesthetic drugs by using a computer-simulated method [5]. Propofol is one of the most commonly used intravenous anesthetics for induction and maintenance of general anesthesia and is usually infused with a TCI device. The real-time monitoring of propofol blood concentration with TCI is calculated and assessed according to pre-set parameters such as gender, age, weight, height, targeted plasma or effect chamber concentration [6]. However, these parameters do not include time-period factors such as during the day or night, which may have effects on the precise implementation of general anesthesia due to circadian differences in pharmacology [4].

Real-time monitoring and modulation of anesthetic depth is necessary for the precise implementation of general anesthesia. Narcotrend is an electroencephalogram (EEG) monitor designed to measure the depth of anesthesia. The Narcotrend index, from 100 (awake) to 0 (electrical silence), indicates the different depths of anesthesia [5, 7]. However, it remains unclear whether the Narcotrend index under propofol TCI is impacted by circadian rhythms. The purpose of this study was to determine whether there is a difference in Narcotrend index related to the time of day during propofol-TCI anesthesia with the same target-controlled concentration for maintenance of general anesthesia. Furthermore, the variation in propofol TCI concentration related to the time of day under the propofol closed-loop TCI guided by bispectral index (BIS), was used to assess the effects of circadian rhythms on propofol TCI.

## Methods

### Ethical approval and registration

The present study was registered on the ClinicalTrials.gov website (NCT02440269) and was approved by the Medical Ethics Committee of Southwest Hospital of Third Military Medical University (ethics lot number: 2016 Research No.93). All patients provided informed written consent to participate in the study.

### Study design

From June 2016 to August 2018, patients aged 18–60 years old with American Society of Anesthesiologists (ASA) physical status I-II, who underwent laparoscopic surgery for appendicitis, intestinal obstruction, ectopic pregnancy, cholecystitis or liver cancer, were enrolled in this study. Patients were excluded, if they met any of the following criteria: ASA ≥ III, uncontrolled hypertension, pregnancy, severe hepatic and renal dysfunction, history of drug or alcohol abuse, current use of psychotropic medicine, inability to communicate or cooperate before surgery and preoperative critical complications with acute peritonitis or sepsis.

Firstly, 30 patients were sequentially divided into two groups according to the operative time (day or night) using the Narcotrend monitor (MT MonitorTechnik GmbH&Co. KG. Germany). Group ND: Propofol TCI with Narcotrend monitor during the day (8:00–18:00), Group NN: Propofol TCI with Narcotrend monitor during the night (22:00–5:00). After completing the case collection for these two groups with the Narcotrend monitor, the other 30 enrolled patients were sequentially divided into two groups according to operative time (day or night) using closed-loop TCI (Beijing Ideas Co. Ltd. China). Group CLTD: Propofol closed-loop TCI guided by BIS during the day (8:00–18:00), Group CLTN: Propofol closed-loop TCI guided by BIS during the night (22:00–5:00). The period of the day or night referred to in Robinson’s and Scavone’s studies [8, 9], were adjusted and narrowed to a shorter time frame, in order to avoid the potential influence of the time boundary between day and night.

### Anesthetic procedure

Patients did not receive pre-anesthetic medication. Non-invasive blood pressure, pulse oximetry, electrocardiogram, body temperature and end-respiratory carbon dioxide concentration were monitored for all patients. Narcotrend and BIS were used respectively for TCI with Narcotrend monitor and Close loop TCI groups mentioned previously.

Oxygen was inhaled at a flow rate of 5L/min for 3 min to remove pulmonary nitrogen. To induce general anesthesia in group ND and NN, midazolam 0.05 mg/kg (Renfu Pharmaceutical Co., Ltd., Yichang, China), sufentanil 0.30 μg/kg (Renfu Pharmaceutical Co., Ltd., Yichang, China), etomidate 0.30 mg/kg (Renfu Pharmaceutical Co., Ltd., Yichang, China) and cisatracurium 0.15 mg/kg (Dongying, Pharmaceutical, Nanjing, China) were used. The anesthetic depth was monitored by the Narcotrend (MT MonitorTechnik GmbH&Co. KG. Germany). The anesthetics used in group CLTD and CLTN for induction, intubation and maintenance included midazolam 0.05 mg/kg, sufentanil 0.30 μg/kg, and cisatracurium 0.15 mg/kg, followed by propofol with closed-loop TCI guided by BIS with a 45–55 target value (Beijing Ideas Co. Ltd. China).

Following endotracheal intubation, the lungs were mechanically ventilated with 50% oxygen combined with 50% air to maintain the pulse oximetry at 95–100% and PetCO_2_ at 30–40 mmHg. The patients in group ND and NN received TCI of propofol (2.5 μg/ml) and remifentanil (3.0 ng/ml). Patients in group CLTD and CLTN received the propofol closed-loop target-controlled infusion and TCI of remifentanil (3.0 ng/ml) with BIS values of 45–55. Cisatracurium was administrated intermittently via single dose intravenous injection guided by train-of-four stimulation (TOF) monitoring in all groups.

Heart rate (HR) and mean arterial pressure (MAP) were maintained within 20% of baseline values through using cardiovascular drugs. When HR < 40 bpm or MAP < 65 mmHg occurred, atropine (0.5 mg/bolus) or ephedrine (10 mg/bolus) was administered respectively. The fluid volume was supplemented according to the basic principles of intraoperative rehydration. The patients’ body temperatures were maintained within 36–37 °C through heat preservation or fluid heating. The anesthetic depth was monitored closely using the Narcotrend in group ND and NN. If the Narcotrend index was below 20 or above 60, the TCI settings (propofol 2.5 μg/ml and remifentanil 3.0 ng/ml) were adjusted to avoid too deep anesthesia or intraoperative awareness, and these cases were withdrawn from the research protocol. Propofol TCI concentration variations were recorded in group CLTD and CLTN.

### Measurements

The primary outcome of this study was to compare the variations in Narcotrend index between group ND and NN. The comparisons of MAP, HR between group ND and NN, as well as the comparisons of propofol TCI concentrations, MAP and HR between group CLTD and CLTN, were secondary outcomes.

### Data collection

The Narcotrend index, MAP and HR were recorded in Group ND and NN at the following time points, T_1_ (5 min before induction), T_2_ (beginning of mechanical ventilation), T_3_ (mechanical ventilation for 5 min), T_4_ (beginning of operation), T_5_ (operation for 10 min), T_6_ (operation for 30 min), T_7_ (end of operation). The propofol TCI concentrations, MAP and HR were recorded in Group CLTD and CLTN at the following time points: T_1_ (5 min after induction), T_2_ (beginning of ventilation), T_3_ (mechanical ventilation for 5 min), and T_4_ (beginning of operation), T_5_ (operation for 10 min), T_6_ (operation for 30 min), and T_7_ (end of operation).

Patient demographics and surgical characteristics, including duration of surgery, time to extubation, length of PACU stay, intraoperative administration of propofol, remifentanil and cisatracurium, fluid volumes, blood loss, urine output, and ephedrine or atropine use, were recorded.

### Statistical analysis

PASS 11.0 (NCSS, Kaysville, UT, USA) was used for sample size analysis. Preliminary investigation indicated that the average value of the Narcotrend index, expressed by mean ± standard deviation (SD), were respectively 49.8 ± 9.3 and 30.7 ± 5.2 in the day and night groups throughout surgery (from T_2_ to T_7_). It revealed that a sample size of four patients per group would detect a significant difference with power of 80% and an α coefficient of 0.05. For the supplementary pre-experiments, the average concentration of the propofol closed-loop TCI guided by BIS were respectively 2.87 ± 0.3 μg/ml and 2.47 ± 0.2 μg/ml (mean ± SD) in the day and night groups throughout surgery (from T_4_ to T_7_). It was calculated that at least seven cases were needed in each group. Considering that there may be cases of dropout or loss of follow-up during the clinical trial, we decided to enlarge the sample size of each group to fifteen cases.

Statistical analyses were performed using SPSS software (version 19.0; IBM, Armonk, NY, USA). The Kolmogorov–Smirnov test was used to evaluate the normal distribution of continuous data. The normally distributed variables were presented as the mean ± SD. The comparisons of Narcotrend index between groups ND and NN and propofol infusion concentrations between groups CLTD and CLTN were completed using a repeated measurement ANOVA with Bonferroni correction. The data for demographics and surgical characteristics, with normal distribution, were analyzed using an independent samples t-test. The non-normally distributed variables such as gender, ephedrine or atropine use, expressed by ratio, were compared using Fisher’s exact test. *P* values < 0.05 were considered statistically significant.

## Results

### Patient characteristics

**Fig. 1:**
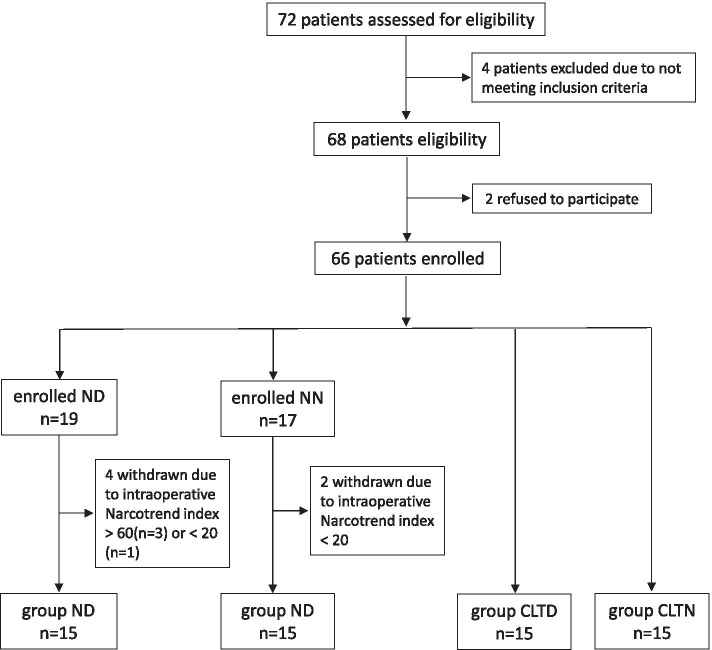
CONSORT flow diagram

A total of 72 patients were evaluated, 68 were identified to be eligible and 4 failed to meet the inclusion criteria (due to ASA ≥ III, cardiopulmonary or renal diseases). Two patients refused to participate in the study. Sixty six patients were enrolled in the protocol. The randomized method was not used for grouping because of the unpredictability of the schedule for laparoscopic surgery at night. Four cases were removed from the enrolled group ND as the intraoperative Narcotrend index was above 60 (n = 3) or below 20 (n = 1). Two cases were removed from the enrolled NN group due to the Narcotrend index falling below 20 intraoperatively. Finally, each group contained 15 cases (Fig. 1). Patient demographics and surgical characteristics are shown in Table 1.

**Table 1:** Demographics and surgical characteristics

Parameters	**Narcotrend (n = 30)**		***P* values**	**CLT (n = 30)**		***P* values**
Group	**ND**	**NN**		**CLTD**	**CLTN**	
Gender (male/female)	4/11	5/10	0.690	6/9	5/10	0.705
Age (years)	39.4 ± 5.7	44.1 ± 4.6	0.283	39.1 ± 4.3	41 ± 3.7	0.200
BMI (kg/m^2^)	20.2 ± 1.4	21.5 ± 2.3	0.088	23.4 ± 1.6	23.4 ± 2.2	0.992
Duration of surgery (min)	235.5 ± 99.4	107.6 ± 26.9	0.000	103.4 ± 30.2	92.9 ± 19.0	0.332
Extubation time (min)	41.2 ± 22.3	10.3 ± 14.0	0.000	7.5 ± 9.9	6.1 ± 6.5	0.698
PACU stay (min)	74.3 ± 33.2	42.4 ± 19.0	0.000	29.9 ± 7.3	36.4 ± 11.5	0.113
Intraoperative propofol (mg/kg)	21.9 ± 6.1	10.1 ± 2.8	0.000	11.5 ± 4.3	8.4 ± 1.0	0.022
Intraoperative remifentanil (mg/kg)	0.03 ± 0.01	0.02 ± 0.01	0.003	0.02 ± 0.003	0.02 ± 0.004	0.182
Intraoperative cisatracurium (mg/kg)	0.8 ± 0.4	0.3 ± 0.1	0.003	0.31 ± 0.11	0.32 ± 0.10	0.800
Fluid infused (ml/hr)	593.1 ± 206.6	707.4 ± 335.3	0.334	568.6 ± 241.4	705.6 ± 314.0	0.189
Blood loss (ml/kg)	5.0 ± 4.8	0.8 ± 0.5	0.006	1.4 ± 1.7	1.4 ± 1.4	0.989
Urine output (ml/kg/hr)	2.0 ± 0.9	2.6 ± 1.5	0.220	0.8 ± 1.0	1.5 ± 0.8	0.037
Ephedrine use(n)	6(40%)	2(13.3%)	0.278	1(6.7%)	3(20%)	0.283
Atropine use(n)	1(6.7%)	0(0)	0.309	0(0)	0(0)	/

***Note***: Data are presented as total number (%), mean ± standard deviation

***Abbreviations***: *ND* Narcotrend monitor during the day (8:00–18:00), *NN* Narcotrend monitor during the night (22:00–5:00), *CLT* Closed-loop TCI, *CLTD* Closed-loop TCI during the day (8:00–18:00), *CLTN* Closed-loop TCI during the night (22:00–5:00), *BMI* Body mass index

### Anesthetic depth indicated by Narcotrend index in group ND and NN

**Fig. 2:**
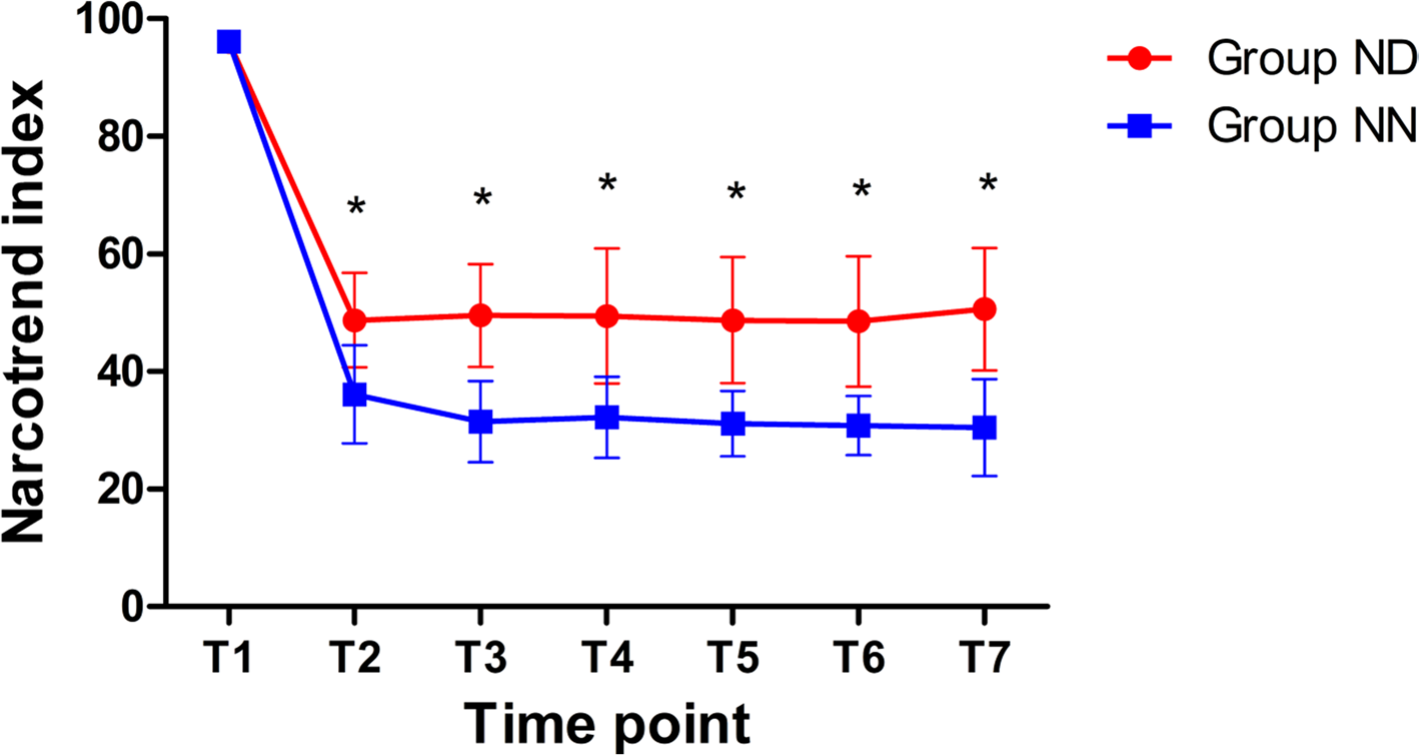
Variations of Narcotrend index in group ND and NN. Note: Data are presented as mean ± standard deviation. *, compared with the Narcotrend index in the group NN at each time point, *p* < 0.05. Abbreviations: ND, Narcotrend monitor during the day (8:00–18:00), NN: Narcotrend monitor during the night (22:00–5:00). T_1_ (5 min before induction), T_2_ (beginning of mechanical ventilation), T_3_ (mechanical ventilation for 5 min), T_4_ (beginning of operation), T_5_ (operation for 10 min), T_6_ (operation for 30 min), T_7_ (end of operation)

At T_1_, the Narcotrend index of group ND (96.07 ± 0.9, 95%CI [95.55, 96.58]) and NN (96.13 ± 0.9, 95%CI [95.62, 96.65]) were both 100 (*p* = 0.853). After anesthetic induction (T_2_), the Narcotrend index dropped to 48.7 ± 8.1(95%CI [44.40, 53.07], *p* < 0.001) in group ND and 36.1 ± 8.3 (95%CI [31.80, 40.47], *p* < 0.001) in NN, respectively. The Narcotrend index values were higher in group ND, compared with group NN, from T_2_ to T_7_ (*p* < 0.001) (Fig. 2).

### Propofol TCI concentrations in group CLTD and CLTN

**Fig. 3:**
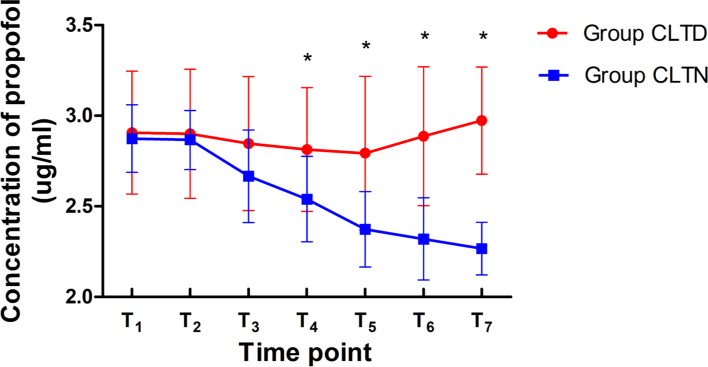
Variations of Targeted concentrations of propofol in group CLTD and CLTN. Note: Data are presented as mean ± standard deviation. *, compared with the concentration of propofol in the group CLTN at each time point, *p* < 0.05. Abbreviations: CLT, Closed-loop TCI. CLTD, Closed-loop TCI during the day (8:00–18:00). CLTN, Closed-loop TCI during the night (22:00–5:00). T_1_ (5 min after induction), T_2_ (beginning of ventilation), T_3_ (mechanical ventilation for 5 min), and T_4_ (beginning of operation), T_5_ (operation for 10 min), T_6_ (operation for 30 min) and T_7_ (end of operation)

At T_1_, T_2_ and T_3_, the differences in TCI concentrations of propofol between group CLTD and CLTN were not statistically different (*p* = 0.741, *p* = 0.744, *p* = 0.132). The propofol TCI concentrations dropped to 2.81 ± 0.3 (95%CI [2.66, 2.97], *p* = 0.017) in group CLTD and 2.54 ± 0.2 (95%CI [2.39, 2.70], *p* < 0.001) in CLTN respectively after beginning the operation (T_4_). The propofol TCI concentrations were higher in group CLTD, compared to those in CLTN from T_4_ to T_7_ (*p* < 0.05) (Fig. 3).

### MAP and HR comparison

MAP and HR in group NN were lower than those in group ND from T_1_ to T_6_. (Table 2). In group CLTD and CLTN, HR and MAP differences were not statistically different at any time point (Table 3).

**Table 2:** Mean arterial pressure and heart rate in group ND and NN

		T_1_	T_2_	T_3_	T_4_	T_5_	T_6_	T_7_
(mmHg)	ND	81 ± 7	72 ± 4	74 ± 5	80 ± 3	77 ± 6	72 ± 5	75 ± 6
	NN	78 ± 9	56 ± 5*	58 ± 6*	62 ± 4*	58 ± 4*	55 ± 3*	57 ± 4*
HR (beats/min)	ND	79 ± 9	75 ± 4	80 ± 8	76 ± 9	75 ± 9	79 ± 5	75 ± 9
	NN	80 ± 8	62 ± 3*	66 ± 5*	59 ± 8*	57 ± 5*	60 ± 3*	62 ± 6*

***Notes*:** Data are presented as mean ± standard deviation. *p < 0.05, vs ND

***Abbreviations*:***ND* Narcotrend monitor during the day (8:00–18:00), *NN* Narcotrend monitor during the night (22:00–5:00). T_1_ (5 min before induction), T_2_ (beginning of mechanical ventilation), T_3_ (mechanical ventilation for 5 min), T_4_ (beginning of operation), T_5_ (operation for 10 min), T_6_ (operation for 30 min), T_7_ (end of operation)

**Table 3:** Mean arterial pressure and heart rate in group CLTD and CLTN

		T_1_	T_2_	T_3_	T_4_	T_5_	T_6_	T_7_
(mmHg)	CLTD	64 ± 2	65 ± 2	64 ± 5	71 ± 5	70 ± 4	71 ± 3	67 ± 4
	CLTN	63 ± 2	64 ± 3	63 ± 3	69 ± 5	71 ± 2	70 ± 4	70 ± 4
HR (beats/min)	CLTD	71 ± 3	71 ± 2	72 ± 3	63 ± 2	64 ± 4	73 ± 2	70 ± 2
	CLTN	69 ± 5	68 ± 3	69 ± 5	59 ± 8	59 ± 6	64 ± 7	68 ± 3

***Notes***: Data are presented as mean ± standard deviation

***Abbreviations***: *CLTD* Closed-loop TCI during the day (8:00–18:00), *CLTN* Closed-loop TCI during the night (22:00–5:00). T_1_ (5 min after induction), T_2_ (beginning of ventilation), T_3_ (mechanical ventilation for 5 min), T_4_ (beginning of operation), T_5_ (operation for 10 min), T_6_ (operation for 30 min) and T_7_ (end of operation)

## Discussion

This study demonstrated that the depth of anesthesia at night was deeper than that in the day when using the same propofol TCI model, under the Narcotrend monitor. The blood concentrations of propofol-TCI were lower at night, compared to those in the day, to achieve a similar depth of anesthesia. Moreover, MAP and HR during general anesthesia at night were lower overall, compared to those receiving general anesthesia in the day, despite using the same propofol TCI. These results suggest that the depth of propofol anesthesia may be impacted by the circadian rhythms.

The results from the present study are consistent with previous studies about the effects of circadian rhythms on drug metabolism in the human body [10, 11]. The possible reasons include variations of hepatic drug metabolism capacity, changes in circadian expression of central nervous inhibitory receptors and the effects of the hypothalamic suprachiasmatic nucleus (SCN) biological clock on anesthetic efficacy, by regulating the release of many internal hormones and transmitters [1]. Firstly, propofol has a very high hepatic extraction ratio, and liver blood flow has a great influence on its metabolism. The hepatic blood flow varies significantly during the day and night, which also impacts the pharmacokinetics of drugs [12]. Additionally, the expression and activity of hepatic cytochrome P450 monooxygenase, which is responsible for the metabolism of most drugs including propofol, may change due to circadian rhythms [13, 14]. Hepatic cytochrome P450 monooxygenase increased at night (active phase) and decreased during the day (rest phase) in a rat model [15]. Secondly, the SCN consists mainly of γ-aminobutyric acid (GABA)-ergic neurons, whose transmitter plays an important role in regulating the circadian clock [16]. GABA and its gated Cl^−^ channel complexes are also the sensitive targets of many anesthetics. General anesthetics act on the brain to produce sedative and hypnotic effects, as well as regulate the biological clock. A recent study showed that GABA receptor expression reaches peak levels in the cerebral cortex during the night and postsynaptic activity is subsequently enhanced [17]. Thirdly, the SCN clock is about 24 h, which is called the free operating period and constitutes the master endogenous biological clock. The circadian clock of the SCN can be influenced by various external "zeitgebers", and its rhythm can be modulated by both natural factors (light, ambient temperature etc.) and artificial factors (drugs etc.). By regulating the release of many hormones and transmitters, including melatonin, cortisol and insulin, the SCN biological clock can affect the phase of the biological clock in peripheral tissues (such as liver and heart), and achieve rhythmic control of life activities such as metabolism, thus affecting behavioral or physiological activities [1]. Evidence from animal experiments suggests that multiple general anesthesia drugs can alter the expression of clock genes [18]. In addition, propofol could increase the plasma melatonin concentration and the interaction between the two substances increases the effect of general anesthetic drugs during the rest phase (day time, for a rat) [3, 19]. Therefore, the pharmacokinetics and pharmacodynamics of propofol may be different when used during the day or night.

The Narcotrend is an EEG monitor designed to measure the depth of anesthesia and is widely used in clinical anesthesia [7, 20]. The Narcotrend algorithm is based on pattern recognition of the raw electroencephalogram (EEG) and classifies the EEG traces into different stages from A (awake, values from 100 to 95) to F (increasing burst suppression down to electrical silence, values from 12 to 0). TCI is a computer-controlled infusion that achieves and maintains a pre-set targeted concentration in a ‘body compartment’. The pumps’ microprocessor calculates the amount of drug, given as boluses and infusions, necessary to obtain and maintain the target concentration using a drug specific pharmacokinetic and pharmacodynamic model [21]. TCI systems are used to administer propofol and opioids for intravenous sedation and general anesthesia for millions of patients every year [22]. In this study, a constant TCI of propofol (2.5 μg/ml) and remifentanil (3.0 ng/ml) could maintain the Narcotrend index values between 20 and 60 for most surgical patients. However, overall the Narcotrend index values in the patients at night were significantly lower than those in the patients during the day (Fig. 2), which indicated the same dose of anesthetic might lead to a greater anesthetic depth at night.

Closed-loop or feedback control systems automatically titrate drug administration to reach and maintain a specific clinical effect, for example the hypnotic component of anesthesia. The infusion rates are changed automatically to maintain a specific drug effect. Some controllers are using TCI technology, combined with a BIS guidance feedback system. A multicenter study demonstrated that closed-loop co-administration of propofol and remifentanil guided by BIS could maintain BIS values in predetermined boundaries during general anesthesia better than manual administration [23]. The BIS is a dimensionless numerical scale that measures brain activity and is derived from EEG signal processing techniques that combine bispectral analysis, power spectral analysis, and time domain analysis using a proprietary algorithm [24, 25]. The BIS range of the TCI-CL system is usually set to 45–55 by default, and the range of BIS values for a suitable anesthetic depth is 40–60. In this study, lower TCI concentrations of propofol at night, compared to those used in the day, maintained the same range of BIS values for the operation (Fig. 3).

It can be inferred from the present study that the required infusion concentration of propofol for general anesthesia during the night should be reduced, compared to that which is used during the day. However, the pre-set parameters of the TCI system do not contain time period factors. Therefore, it can be suggested that during the day modes should be designed into TCI systems for more appropriate anesthetic depth modulation in the future, especially in the absence of BIS guided closed-loop feedback support.

There are some limitations in this study. Firstly, opioid drugs included in the study can affect the depth of propofol anesthesia. The anesthetic depth shown by the Narcotrend index or BIS value, was achieved by the combined action of propofol and remifentanil, even when a uniform TCI concentration of remifentanil is maintained. Secondly, the laparoscopic surgery selected in this study has little influence on the overall physiological function of the patient. Whether the findings can be generalized to other types of surgeries needs further investigation for confirmation. Thirdly, 2 patients undergoing laparoscopic hepatectomy and 6 patients undergoing laparoscopic gynecological malignancies surgeries, a long-term operation, were included in the group ND. This caused many baseline values of surgical characteristics, including recovery time, consumption of intraoperative anesthetics and total fluid volume, to be significantly different. Under these limitations, we were unable to calculate the changes in MAP and HR from baseline, and thus were unable to account for the natural dip in BP across the night that was observed in most individuals. This potential bias could significantly impact both the primary and secondary outcome measurements and limits the extrapolations that can be made from comparisons of the day and night patient groups.

## Conclusions

In summary, circadian rhythms can influence the depth of general anesthesia and drug infusion concentrations during target-controlled infusions of propofol. In clinical practice, the propofol TCI concentration should be appropriately reduced when general anesthesia is implemented at night.

## Data Availability

The datasets used and analyzed during the current study are available from the corresponding author on reasonable request.

## Abbreviations

TCI: Target-controlled infusion
ND: Narcotrend monitor during the day (8:00–18:00)
NN: Narcotrend monitor during the night (22:00–5:00)
CLT: Closed-loop TCI
CLTD: Closed-loop TCI during the day (8:00–18:00)
CLTN: Closed-loop TCI during the night (22:00–5:00)
BMI: Body mass index
MAP: Mean arterial pressure
HR: Heart rate
ICU: Intensive care unit
ASA: American Society of Anesthesiologists
BIS: Bispectral index
PetCO_2_: End-tidal carbon dioxide
TOF: Train-of-four stimulation
PACU: Post anesthesia care unit
